# Exploring the Process of Policy Overreaction: The COVID-19 Lockdown Decisions

**DOI:** 10.1177/10564926221082494

**Published:** 2022-03-07

**Authors:** Taieb Hafsi, Sofiane Baba

**Affiliations:** 1 Holder of the Strategy and Society Chair, HEC Montréal; 2 7321 Université de Sherbrooke

**Keywords:** decisions under risk/uncertainty, COVID-19‌, affect/emotions, public management, decision-making, team/organization

## Abstract

Policy overreaction is a common phenomenon, especially in complex and emergency situations where politicians are led to make decisions fast. In these emergency decisions, emotions run generally high and cognitive processes are often impaired. The conditions of policy overreaction are in place as emotions overwhelm decision makers’ rational processes. Drawing on the response patterns of three countries to the COVID-19 pandemic, we develop a process model of policy overreaction which describes the effects of negative emotions and institutional isomorphism on policy decision-making. Our model highlights four critical stages: negative emotions buildup, propagation of fear, isomorphic decision-making, and leading to an intractable crisis. This article shows precisely how the cascading effect of negative emotions, particularly fear, is contagious and spreads to generate crowd effects, which bend considerably policy makers’ ability to make rational decisions. Our theory provides a better understanding of the process by which policy overreaction takes place.

## Introduction

Policies determine how governments respond to and manage crisis events. When events are momentous, how measured and effective reactions are is often of great societal consequences (Dror, 1983). For example, in reaction to the installation of nuclear missiles in Cuba, the U.S. government has succeeded in securing the removal of the missile site and averting a nuclear confrontation (Allison, 1971). In contrast, the government's response to the N.Y. Twin Towers terrorist attack, on September 11, 2001, led to devastating wars in Afghanistan and Iraq, countries later to be widely seen as unrelated to the event (see Bolger, 2014; Lansford, 2012), thus damaging the nation's well-being and international status^1^ (Sanger & Shear, 2021; Tripathi, 2010). More generally, policies in response to major events, whether natural or man-made, could have considerable consequences for the welfare of entire populations and sometimes for humanity as a whole (Desch, 2008; Maor, 2019; Muller, 2021).Yet, overreaction is quite common (Jones et al., 2014; Maor, 2018; Muller, 2021).

The SARS-CoV-2 infection started on December 11, 2019, in the city of Wuhan in China. Wuhan is in the heart of mainland China, at the confluence of the Yangtse River and its largest tributary, the Hanshui. The city was totally locked down, and the Chinese government provided a show of how to deal with a dangerous pandemic, with precise organization and impressive resources. China was the birthplace of the first coronavirus, the SARS-CoV-1, in 2002–2003. The Chinese authorities and scientists were supposed to know the virus type. It was therefore surprising to see the Chinese government reacting with such a massive force. Western governments’ assumption was that China encountered a truly dangerous coronavirus form. In late February 2020, Europeans were alarmed by an apparent Italian public health inability to handle a deadly spread of the same virus in the Bergamo region^3^. On March 11, 2020, the World Health Organization declared the SARS-CoV-2 a pandemic.

As a result, in quick sequence, starting early March, the governments of most Western countries responded to the threat of “a dangerous virus-induced disease” with massive lockdowns. They were obliged to also provide support to economic activities, whose staggering direct costs were estimated to be at least 16 trillion dollars in the United States alone (Cutler & Summers, 2020; Powell, 2020). Yet, despite furious debates between scientists, about how dangerous and lethal was the viral infection or even how to treat it, no clear conclusions can be stated (Cheung & Parent, 2021; Mucchielli, 2020; Raoult, 2021; Vaja et al., 2020). Rather, compared to previous pandemics, the fatalities appear to be well below averages (Garber, 2021; Wong et al., 2013). Did Western governments overreact? Judging by the cost and turmoil generated, and the uncertainty cast on the recovery of the world economy, the lockdown can be seen as policy overreaction (Bhattacharya & Vermund, 2020).

In his detailed study of the South African response to the SARS2 infection, Muller (2021) argues that overreaction is obvious, when one looks at the details. Quality of available information, emotions involved, and limited effects of the drastic measures used, all point to unjustified extreme policies. Late in 2021, lingering effects of the pandemic and an apparent inability to get rid of the infection despite all efforts point at the limits of radical policies. Amidst the generalized failure to deal with the viral infection, there are, however, two important exceptions: The Chinese ability to stop the infection within 2 months of its appearance, a few flaring episodes notwithstanding, and Sweden's contrarian policy not to enforce a lockdown (Tegnell, 2021), with effects that were at the end similar or better than those of the more extreme policies.

Studies of crises (see Rodríguez et al., 2007) highlight some determinants of extreme behavior. Most of them are related to decision makers’ personal characteristics, their experiences with crises, and their reactions to strong negative emotions. The effects of emotions on decision-making have been widely studied (Andrade & Ariely, 2009; Neumann, 2017). Reason is affected by strong emotions, sometimes with great harm to people and their well-being. Decision makers could lose sight of realities and make harmful hasty decisions.

To design effective responses, we need to go beyond leaders’ characteristics to the systems and processes that give way to the effects of their failings. This was done to explain disasters, but unusual threats such as biological infections are more challenging. There are few empirical studies of how societies do or should respond to broad infection threats. A conceptual piece by Maor and Howlett (2020) suggests such determinants as psychological (e.g., panic, fear, and negative emotions), informal and formal institutional factors (e.g., degree of centralization), and strategic (potential political gains from measures dealing with the pandemic). However, their study provides rationales for determinants but not an integrated theory to explain the why and how.

In this qualitative research paper (Edmondson & Mcmanus, 2007), we intend to fill that gap with a multiple case design (Eisenhardt & Graebner, 2007). Using a wealth of secondary data about governments’ decisions, we study how policy decisions to lockdown activities in China, France, and Sweden were made in a context of high uncertainty and emergency. This article does not make any value judgments about governments’ protracted handling of the pandemic. The management of the 2020–2021 crisis is not the focus of this paper. Rather, we focus on the initial response to the pandemic, that is, the lockdown in the first months of the pandemic, and how it was fueled by policy overreaction. Specifically, to describe it, we propose a process theory of policymaking under uncertainty and severe time constraints. We suggest that the way policy decision-making is designed, and the context of decisions, could interact with human characteristics in unique ways to explain behavior. In so doing, we contribute to the existing literature on this topic (Desch, 2008; Jones et al., 2014; Maor, 2012, 2018). In a few concluding comments, we make recommendations for policymaking in situations of emergency crises.

## Theory: Policymaking Under Uncertain Circumstances and Severe Time Constraints

In this section, we describe and discuss the first decision-making processes from an organizational and institutional perspective. Second, we address the literature on major policymaking under information uncertainty and urgency. Finally, we conclude our theoretical framing by discussing the role of emotions in decision-making.

### The Decision-Making Process and Institutional Effects

Organizations can modify the rationality of decision makers by influencing the set of premises that they consider. Simon and the decision-making process school (for useful summaries, see Allison, 1971; Bower, 1970; Cyert & March, 1992; Simon, 1997) have suggested that these influences are imbedded in most managerial processes, including (1) authority, (2) organizational loyalty, (3) the criterion of efficiency, and (4) advice and information, and (5) training (Simon, 1997).

Collective decision-making brings to the table the need to reconcile the factors affecting individuals and thus individual preferences themselves, which may increase the level of complexity, reduce the understanding of cause-and-effect relationships, and lead back to random behavior (Cyert & March, 1992). Three important views have dealt with complexity. Allison, in his study of the Cuban missile crisis, proposed that complex decision-making, for example, when the President of the United States has to make policy decisions, is affected by three dominant logics.

The first is the rational model. Decision makers’ logic is to protect or enhance the whole country's well-being. Strategic management and economic rationality provide the tools for such an analysis. The second model is organizational in nature. In complex country states, many different organizations are involved in policy decision-making. Their choices are affected by what Cyert and March (1992) labeled routines or standard operating procedures. The interests pursued are those imposed by the organizations involved. The third is the political model, with decisions dominated by individuals’ preferences, power, and ability to influence others. Thus, the broad policymaking process could be seen as being at the same time rational, organizational, and political. Bower's study of the resource allocation process in large corporations describes a similar model and has been shown to apply to other organizational types, particularly those of the public sector (Hafsi et al., 1987).

If Allison and Bower provided the structure through which decisions are made in large complex organizations, Braybrooke and Lindblom (1963) show how complex policy decisions are made in the United States and why that process generates the best possible decisions. They show that the traditional rational model breaks down in situations of complexity, where cause-and-effect relationships are obscure. Using the economic welfare function as an example, they show how attempts to capture complexity through a “synoptic” model have failed. They show that policy decisions are made through a “disjointed incrementalism” process. When complex decisions should be made, policy formulation is open to input and influence from groups or individuals. This decentralized disjointed process, they argue, is likely to lead to the best study for the problems at hand because all or most interested parties are motivated to articulate and highlight their perspectives. But a decision must be made, and any choice is unlikely to satisfy everybody. There will be winners and losers. Why would losers come back at the next policy decision? According to Braybrooke and Lindblom, it is because most policy decisions in the United States are incremental. The losers never lose entirely. They remain motivated to keep fighting and maintain a quality study of policy implications.

Thus, to deal with complexity, which tends to generate overwhelming uncertainty and obscure cause-effect relationships, a fragmented, step-by-step process, through which learning takes place, and adjustments are made, is best. This seems to be the case for the early periods of a pandemic when viruses are unknown and health effects unclear.

Individual and organizational decisions at the state level are also influenced by unseen factors, commonly referred to as “institutions” (North, 2010). In addition to broad rules and procedures, institutions include norms of behavior, whether professional or general, and influences shaped by education and culture. Rules and procedures are clearly seen in the pandemic situation. Governments change rules to control population behavior. Organized authority, such as the police departments, and other vigilantes, could be summoned to ensure enforcement, using coercion, the threat of severe penalties.

Norms of behavior are unwritten rules that sometimes determine decisions by shaping the way individuals and organizations reason and their specific rationality (Scott, 2014; Selznick, 1957). Oliver (1997) described how norms and cognitive cultural influences bend individual and organizational rationality. Institutions shape behavior, sometimes beyond the expected instrumental rationality. They have a homogenizing effect. Thus, organizations and individuals are led to isomorphic behavior, imitating what others do or expect them to do. DiMaggio and Powell (1983) have made a case for this tendency to imitate others, and Oliver (1997) has shown that this affects deeply individual, organizational, and field-level critical decisions.

### Major Policymaking Under Information Uncertainty and Perceived Emergency

The coronavirus is a well-known form of viral infection (Weiss & Navas-Martin, 2005). Depending on many factors, most importantly individual health conditions, it may have mild or severe health effects (Raoult, 2021). The SARS-Cov-2 virus was an unknown variety. Early information provided by Chinese health authorities indicated similarity with SARS-CoV-1. The information available was sketchy (Graham-Harrison & McKie, 2020). On the one hand, some virus experts (biologists and epidemiologists) provided highly sophisticated descriptions of the general characteristics of coronaviruses, which, although accurate, did not explain why this was a dangerous virus and how to deal with it in concrete public health terms. On the other hand, simplistic projections of how the disease could spread and hospitalizations explode, provided catastrophic predictions of overwhelmed hospitals and mass casualties. Researchers’ specialized presentations trickled into mass communication and became the subject of public debate with limited attention to how the research was built, and its real meaning, thus increasing information distortion (Mercola & Cummins, 2021; Raoult, 2021).

A crowd effect and perceptions of danger among policymakers soon became overwhelming. The media, in particular social media, compared with previous dangerous pandemics, amplified the perception and generalized the sense of danger. Fear took over, as in a crowd effect (Canetti, 1984). In this generalized lack of precise and careful information, governments were pressured to act and most followed suit, doing basically what the Chinese government appeared to have done. They locked down entire countries, stopping the functioning of most activities, encouraging people to stay home, and believing that henceforth the disease would go away. The conditions for policy overreaction were in place.

When emotions are high, “extreme predictions and a willingness to predict rare events from weak evidence are common” (Kahneman, 2011, p. 194). Maor (2012) argued that policy makers’ overconfidence, in particular an overestimation of their ability to control the pandemic, their belief that they are the best able to do it (overplacement), and that they have accurate information (overprecision), leads to the temptation to take what appears to be decisive actions to rid the masses of the dangerous challenge. This is coherent with Kahneman's (2011, p. 195) argument that overconfidence and optimistic bias “may well be the most significant of cognitive biases.” The literature confirms contagion, and the extent of individual and group confidence is relatively the same (Polansky et al., 1950; Puncochar & Fox, 2004). Therefore organizations and institutions “are not likely to be able to protect against the enthusiasm and/or misjudgment of policy-makers.” (Maor, 2012, p. 240)

Groupthink is another key element affecting decision-making and individuals’ ability to discuss, debate, and adjust their thinking. Originally coined by Janis (1972), groupthink means a “mode of thinking that people engage in when they are deeply involved in a cohesive in-group when members’ strivings for unanimity override their motivation to realistically appraise alternative courses of action” (Janis, 1982, p. 9). According to Janis (1972, p. 245), “illusions of invulnerability,” “collective rationalisation,” “illusion of unanimity,” “self-censorship,” and “belief in inherent morality” are groupthink characteristics. In situations of power asymmetry, “strategies of realism or denial will ‘trickle down’ the hierarchy so that subordinates will in effect take their beliefs from the leader.” (Bénabou, 2013, p. 2).

When dealing with severe pandemics, two options are generally available to policy makers, suppression/containment and mitigation (Ferguson et al., 2020). The first, a severe intervention to limit the infection to a number of people, was used for the Ebola epidemic. The second is intended to reduce the speed with which the infection spreads, not the number of people infected. In popular parlance, officials talk of “flattening the curve.” (Market et al., 2020). It involves managing large populations and was widely adopted for the SARS-CoV-2 pandemic. The challenges of managing populations’ behavior are considerable and open to excesses.

Muller (2021) studied with precise information details the South African decisions in response to SARS-CoV-2. He argues that government officials have been excessive and unscientific in their efforts to convince the population to comply. For him, policy makers engaged in *performative scientism*, through which they “seek credibility for their approach by performing excessive deference to what they believe to be ‘science’” (p. 1) Haack (2012, p. 75) defined scientism as an “inappropriately deferential attitude to science” (Haack, 2012, p. 75). According to Muller (2021, p. 1), “the South African government's emphasis on an ostensibly scientific approach has been extreme and simultaneously shielded it from necessary scrutiny in a decidedly unscientific manner” (p. 2). In particular, the focus solely on medical science, in a multidimensional crisis, was bound to lead to mistakes. He concludes with three major lessons: (1) treat “certitude” with skepticism; (2) consider external validity with caution because many endogeneity issues could obscure cause-effect relationships; (3) look at others’ formulas as perhaps context-specific.

In their study of the Netherlands experience with the pandemic, Janssen and Van der Voort (2020) emphasize the need to distinguish between agility and adaptability. The first, valued by most governments, can come at the expense of the ability to adapt, especially in the long run. They conclude that “adaptive governance requires a high tolerance for paradox. It involves both rapid and sound analysis for decisions. It requires both centralized and decentralized mechanisms, innovation and bureaucracy.” (p. 6) Thus, confirming the consensus among many studies that a multidimensional crisis is complex and should be treated with a requisite variety of perspectives (Braybrooke & Lindblom, 1963; Geyer & Cairney, 2015; Mueller, 2020), instead of simply a medical viewpoint.

The willingness of governments to treat fast a complex crisis that requires careful attention is a problem, according to Reicher and Stott (2020, p. 702). Looking into the French and British experience, they warn: “unless more steps are taken to make it possible for those in precarious positions to cope with lockdown and other measures; unless the focus of the police and other agencies is more … on enabling rather than enforcing …, then the precious and fragile social consensus … could always give way to social conflict.”

### Emergency, Uncertainty, and Emotions

Elfenbein (2007) provided a useful literature review in the Academy of Management Annals on the role of emotions in and around organizations. Studies of how emotions affect attitudes, moods, and actions are widespread (Neumann, 2017; Tamir & Bigman, 2018). In particular, emotions affect decision-making which is normal considering that strategy is embedded in every day seemingly mundane interactions (Samra-Fredericks, 2004) and that “emotion plays an important role in these micro-interactions.” (Kouamé & Liu, 2020, p. 9).

Common beliefs are that emotions have a pernicious effect on decision makers’ thinking (Forgas, 2009), and research tend to confirm that. For example, emotions can divert attention and interrupt one's thinking (Beal et al., 2005; Weiss & Cropanzano, 1996). Strong emotions crowd cognitive capacity, in particular attention, memory, and logic (Clore et al., 1994; Schwarz, 1990). This is sometimes useful, when attention is focused on targeted goals, but generally disruptive, distorting our appreciation of decision consequences (Loewenstein & Lerner, 2003). Research provides ample evidence that learning, memory, associations, social judgments, and social interactions are consistent with individuals’ emotions (Clore et al., 1994; Forgas & George, 2001). As an illustration, it has been suggested that positive emotions lead to more optimistic decisions while negative emotions are more likely to generate pessimistic decisions (Fenton-O’Creevy et al., 2011; Mayer & Hanson, 1995).

Negative emotions impact cognition even more, increasing the sense of danger, or emergency, collapsing the time available, and severely disrupting cognition (Andrade & Ariely, 2009; Angie et al., 2011). For instance, when decision makers perceive that there is a danger or an emergency, stress builds in and emotions can take over (Castellnou et al., 2019; Mizrahi et al., 2019). In these conditions, stress and emotions have a deleterious effect on how decisions are made (Maner et al., 2007). In situations of emergency, when stress is high and fear common, the scope of decision-making tends to be narrower (Isen, 2001; Tiedens & Linton, 2001), the analytical process is shortened (Gok & Atsan, 2016), and groupthink affects the ability to challenge assumptions (Chapman, 2006).

Rather than stress itself, the perception of distress appears to compromise judgment (Gillis, 1993). The more perceptions are distorted, the more judgments and problem-solving processes are distorted. In a laboratory study of forest firefighting, individuals under stress focused on generalities, while non-stressed ones relied on in-depth analysis. Kontogiannis and Kossiavelou (1999) confirmed that stress “restricts cue sampling, decreases vigilance, reduces the capacity of working memory, causes premature closure in evaluating alternative options, and results in task shedding.” In studies of underground mine fires, as fear and stress are high, judgment is often based on unclear, faulty, and incomplete information. In these situations, decision makers cannot come up with appropriate responses. Individuals’ age, sex, level of experience, and training with emergencies moderate such a behavior (Galván & McGlennen, 2012; Kontogiannis & Kossiavelou, 1999; Uy & Galván, 2017). The context is also important. In particular, dynamic environments reduce adaptation capacity (Kerstholt, 1994; Kidd et al., 2013).

Taken together, the literature on emotions is rich and multidisciplinary but focused on the main actors. What is less studied in the literature are those complex situations where fear is mostly collective (shared and reinforced along the way) and generated at different levels (e.g., population, media, and politics). We posit that such situations distort decision processes and policy responses. Taken collectively, this literature review leads us to explore: *how can collective fear and its propagation lead to policy overreaction?* In what follows, we focus on how collective and institutional fear influenced early policy responses to SARS-Cov-2.

## Methodology

### Overall Research Approach

Exploratory and phenomenological, this article's qualitative approach allows the study of intertwined processes at the social, organizational, and institutional levels (Patton, 2002; Reay et al., 2019). We adopt a holistic multi-case study (Eisenhardt, 1989), searching for theoretical insights on under-explored phenomena and research questions (Yin, 2003). Multi-case studies “enable collection of comparative data…likely to yield more accurate, generalizable theory than single cases.” (Ozcan & Eisenhardt, 2009, p. 249). This is also consistent with our focus on countries’ policy response differences.

Our empirical setting is made of three countries’ early policy responses (i.e., China, France, and Sweden) to the 2019–2021 SARS-CoV-2 pandemic. In addition, we chose three countries whose approaches to decision-making in dealing with the pandemic were contrasted. Our three fields of inquiry emphasize various analytical dimensions:

1. The base case of China's handling of the SARS-CoV-2 epidemic, emphasizing justifications for decisions made.
2. The initial French decision to lockdown appears with hindsight as one of the most radical in Europe and the World.
3. The Swedish contrarian decision to avoid lockdown and proceed cautiously to deal with the pandemic effects.

### About Our Empirical Context: A Rare Contemporary Extreme Event

We believe that the SARS-CoV-2 pandemic is unique in its own right (see Mercola & Cummins, 2021; Raoult, 2021). The abrupt and unexpected nature of the virus, its rapid spread and evolution, institutionalized fear, and the lack of reliable and stable knowledge given the scientific and medical controversies, make this case a unique empirical opportunity to study policymaking under emergency. Moreover, the SARS-CoV2 infection generated a crisis that lasted more than a year, affected most countries, and disrupted a highly integrated world. The situation and decisions in one country could influence many others. The policy process in any country was therefore faced with overwhelming complexity. Cause-and-effect relationships were very hard to predict, and in such a situation, experimenting can be seen as the rational way to proceed (Braybrooke & Lindblom, 1963). In addition, after over a year of upheavals and haphazard management, populations were fearful, disoriented, and generally dispirited. At the end of 2021, severely disrupted, it was unclear how economies would recover. Optimists emphasize the opportunity to undertake a change in collective behavior and deal with climate change. Pessimists see humanity stepping back to an era of mistrust and confrontation (Reicher & Stott, 2020).

### Data Collection

This paper relies on extensive and varied secondary data available in the public domain. Considering that the amount of information and data on the topic is overwhelming, the challenge was to select the more significant and relevant. We looked for convergence among different sources, keeping in mind the importance of triangulating data to ensure empirical rigor and validity (Flick, 2018; Patton, 2002). Five relevant sources were consulted and analyzed (Table 1), including newspapers, video documentaries, media debates, experts’ interviews, and other materials such as books. This rich, quality data and their analysis ensure finding transferability, in line with the interpretative posture of this research (Lincoln & Guba, 1994). A systematic data collection began during the summer of 2020, then was clarified and completed in March 2021. This provided time to reinterpret the summer data from a new perspective considering that discourse and events evolved fast.

**Table 1.** Empirical Data.

Data source	Type and details	Use in our analysis
Newspapers	Systematic keyword searches were conducted in mainstream international newspapers (The Guardian, Le Monde, The New York Times, The Globe and Mail, and The Epoch Times.) With the help of research assistants, we searched for several keywords (2019–2021 period): “Covid,” “Coronavirus,” “SARS-CoV-2,” “Emotions AND Covid,” and “Covid AND response.” In addition to the two main keywords, we also conducted other individual searches, including the country (e.g., China, France, and Sweden). Overall, over 250 articles have been used to shape our understanding of the phenomenon studied, mostly in English and French.	Newspapers were the main source of data used to develop a chronological understanding of the pandemic, especially with regards to major decisions such as lockdowns. We also heavily used newspapers to delve into the structure underlying the decision-making process of each of our cases, as well as the rhetorical justification of lockdowns. Moreover, newspapers were useful in helping identify key actors involved in nationwide decision-making (e.g., China, France, and Sweden) and their discourses over time.
Video documentaries	Video documentaries were also consulted because they are rich sources of information. Experts from various backgrounds, often with contrasting opinions, took part in these documentaries. Similarly, politicians also spoke to justify their actions, which provided us with relevant data for our study. Over 15 documentaries were analyzed, focusing on how our three contexts (e.g., China, France, and Sweden) dealt with the coronavirus pandemic. These documentaries were in many cases produced by major television channels (e.g., Al Jazeera, BBC, the national networks of the three countries, and Netflix.) The duration of these documentaries ranged between 15 min and 60 min.	Documentaries were mainly useful to consider the multiple perspectives on the pandemic and the means to manage it at the political level (e.g., politicians, scientists, experts, activists, population, and whistleblowers). They were also useful in historically positioning the coronavirus and the reactions of governments to manage them. To some extent, documentaries have helped us better determine the turning points in this controversy, emphasizing critical decisions.
Media debates	Media debates between politicians, journalists, analysts, and experts were analyzed. These debates were mostly watched on English-speaking channels (BBC, CNN International), and on French channels (BFMTV, France 24, TV 5, and LCI). They focused on the pandemic, its scientific dimensions, and the effectiveness of government responses worldwide, including the three countries that constitute our cases.	The debates contributed to our analysis by showing the points of contention on the good and bad moves in the national management of the pandemic. The debates were particularly useful for contrasting the experiences of the three countries studied. Debates also helped in putting into perspective various visions of the management of the pandemic, in particular in connection with the decisions to confine the populations and the vaccination campaigns.
Experts’ interviews	Over 20 h of experts’ interviews were also analyzed, such as Didier Raoult’s (one of the most popular and controversial epidemiologists in the world) videos on his social networks and his multiple interviews on various French channels. Other experts were also listened to, such as those of Johns Hopkins Bloomberg School of Public Health and the New Yorker. Finally, several books on the coronavirus pandemic were also consulted and analyzed (Koley & Dhole, 2021; Rabadan, 2020; Raoult, 2021).	The expert interviews as well as the other sources made it possible to deepen and triangulate the data collected. In particular, they served to deepen specific perspectives by focusing on the discourse of certain highly publicized experts, who have greatly shaped our conception of the pandemic. Academic articles have been helpful in genuinely understanding the state of knowledge on the subject.
Other relevant material	Other sources, including books (Koley and Dhole, 2021; Mercola and Cummins, 2021; Rabadan, 2020; Raoult, 2021; Reiss and Bhakdi, 2020; Schwab and Malleret, 2020) and published peer-reviewed papers (Bacq and Lumpkin, 2020; Maor and Howlett, 2020; Martin et al., 2020; Muller, 2021; Tegnell, 2021; Weiss and Navas-Martin, 2005), have been gathered on the SARS-CoV2 virus and on the management of crises, using ProQuest, a well-known database covering academic and professional publications.	

### Data Analysis

**Figure 1.**
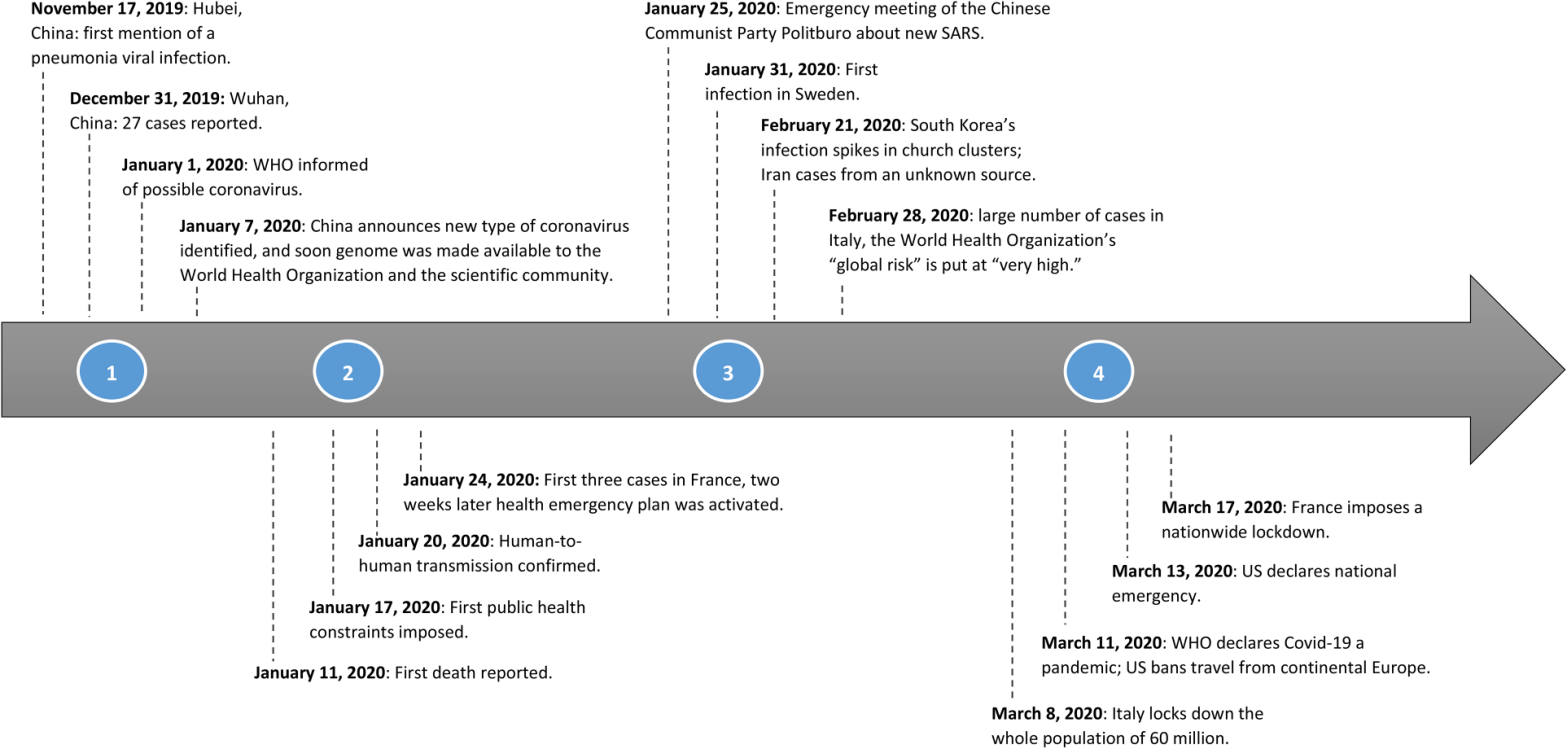
Timeline of COVID-19 outbreak.

We analyzed our extensive data using abductive logic (Timmermans & Tavory, 2012). We engaged in several iterations of analysis, contrasting existing theories with emerging findings from our data. We proceeded in three stages. First, a chronology of policymaking responses was prepared for each of our contexts. These chronologies were particularly interesting for identifying ruptures “of some kind… that is, a surprising break with routine practice” (Sewell, 1996, p. 843). Along with the chronologies (Figure 1), we drafted descriptive “thick narratives” for each case (Geertz, 1973).

Second, we began to make sense of these narratives through an emerging theoretical framework, combining decision-making and emotional theoretical insights. Looking for specific instances where emotions and decision-making were obvious, we realized that Allison's three models generally explained decisions in our cases: rational or strategic, organizational, and political (Allison, 1971). Our analysis thus covers: (1) the rational model as captured by the historical and geopolitical background of the country, in particular perceived outside threats to security and to economic development; (2) the organizational model through those state structural arrangements, which channeled the country's response to the pandemic; and (3) the political model as evoked by the emotional state of key people, in particular leaders and their advisers.

**Figure 2.**
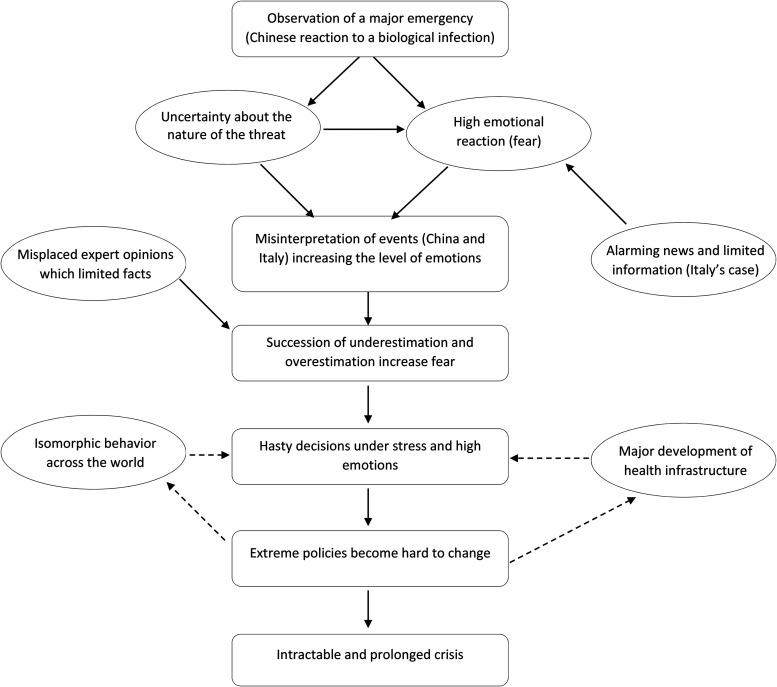
A model of policy overreaction.

Third and lastly, going back and forth between data and initial theoretical insights (Patton, 2002), an emerging process-model of decision-making in the face of danger and emergency emerged. This model has four stages: (1) emotion-building events, (2) fear and its propagation, (3) isomorphic lockdown, and (4) intractable and prolonged crisis. Overall, this data-driven process model explains how decisions are made in extreme situations of uncertainty and emergency. The four stages are the overarching themes of our empirical findings. Each theme is illustrated through vignettes, stories of what happened in the three countries studied. Figure 2 illustrates our model.

## Empirical Findings: A Process Model of Policy Overreaction

Governments’ responses to the pandemic have been isomorphic. Faced with uncertainty, they have all been led to a lockdown and were looking for a common way to end the resulting crisis at the end of the pandemic. Everywhere, policymaking has been derailed by emotion, in particular fear, then dominated by a focus on health science and on the opinions of highly specialized health epidemiologists. Little place has been given to any other considerations. As a result, most decisions have been drastic, with either massive lockdowns or hasty openings. Instead of slow decision-making, more conducive to learning, fast and hurried has been the norm. Using Allison's framework, the political model has taken over, with politicians’ emotions, interests, and preferences taking precedence over facts. Effects have been magnified by the organizational model, the way bureaucratic organizations enforced the decisions made, and justified them using “performative scientism” (Muller, 2021), with apparently rational health-based arguments. This sketches our proposed model, which is now described and discussed through Vignettes 1 to 5. Table 2 offers empirical details supporting our analysis.

**Table 2.** Empirical Illustration of the Evolution of the Pandemic in China, France, and Sweden.

	Events pre-lockdown and government justifications	Structure to build the decision to lockdown	Knowledge about virus	Emotions and misperceptions as expressed by decision makers
China	China presents a significant increase of cases in January 2020, and people-to-people transmission is confirmed. On January 19, around 40,000 families gathered in a mass Lunar New Year banquet, where many people got infected. With the Chinese New Year approaching, authorities close Wuhan City on January 23, with then 830 cumulative cases: With a mass movement of people in perspective, if there is no lockdown of Wuhan, other cities will fall under a huge wave of cases and have an impact on people’s safety, economic development, and social stability. From January 24 to 30, the rest of the country celebrates the Lunar New Year holiday, so hundreds of millions of people travel around the country to visit their families and friends. When the cumulative cases hit 7,711, the authorities imposed a nationwide lockdown effective on January 29. Xi said making the decision required enormous political courage: “But time calls for resolute action. Otherwise, there would be trouble” he said. Xi also stressed the importance of putting people's health and lives, as well as their interests, at the forefront. . He said all prevention and control measures by the CPC Central Committee were taken with the primary objective of preventing infections among the people and saving lives.	**Communist Party of China (CPC)** General Secretary Xi Jinping lead the COVID-19 response. Placing great importance to COVID-19 prevention and control, Xi Jinping took full responsibility over the control efforts from the very beginning. **Central Leading Group for COVID-19 Prevention and Control (CLG)** The strategic decisions around China’s response were made centrally by the CLG. **Central Steering Group for COVID-19 Prevention and Control (CSG)** Formed to supervise the anti-pandemic campaign in Hubei Province, the COVID-19 epicenter. **State Council Joint Mechanism for COVID-19 Prevention and Control** “Enlisting all central ministries related to a public health emergency”, it “aimed to break down interdepartmental barriers” to facilitate policy coordination (He et al., 2020, p. 246). **Local authorities and other stakeholders** The central command structure was replicated in all local governments, “which quickly set up local command headquarters for COVID-19 prevention and control” (He et al., 2020, p. 246) In general, an impressive level of involvement on the part of most authorities, with local initiatives and a close monitoring from the top.	On January 2, a leading group was formed by the NHC and came up with a set of guidelines on discovery, diagnosis, and quarantine to prevent and control this unknown disease. On January 7, China CDC had successively isolated the first strain of the novel coronavirus, linking it to SARS and MERS viruses. NHC identified the new coronavirus as the cause of the epidemic. Authorities made the genome of the new virus publicly available on January 10. China CDC also shared the specific primers and probes for the detection of the virus with the WHO. Human-to-human transmission has been confirmed by the NHC.	China's government discourse often includes key terms referring to war, such as total war, battle of annihilation, and people's war. Community mobilization prone by the anti-pandemic campaign was encouraged through a vision of the battle between the virus and humankind. The use of war narratives in China's civilian affairs is widespread due to the cultural past of the communist armed struggle in the revolutionary era. War narratives also appeal for civilian behavioral compliance with coercive measures because of the emotional mobilization caused by the sense of urgency. “While similar narratives are also found in the speeches of political leaders and official documents of other countries... such extensive use in China is remarkable.” (He et al. 2020, pp. 251-252). Possibility that China may have looked at viruses as a vehicle for biological war.
				
France	Italy experienced its first lockdown during the third week of February 2020. Italy is put under total lockdown on March 13, 2020. As of March 14, 2020, the stage 3 of the epidemic is engaged in France. The French government imposed the lockdown on March 17, 2020. This decision was taken in the light of a dramatic apprehension of an imminent wave of cases and the blatant lack of preparation of the French health system, including limited beds, masks, and testing capacity. At the time, lockdown was seen as the only possible solution, rather than the best one, and was heroically promoted because any other option would cost too many lives. In the world, as of March 15, 2020: 215,998 confirmed cases since the beginning of the epidemic The majority of new confirmed cases are now being reported outside China and especially in Europe where transmission has intensified in recent weeks. In France, as of March 15, 2020: 4,934 confirmed cases since the beginning of the epidemic There is a doubling of the number of new cases declared between March 13 and 15, indicating an intensification of transmission throughout the country. Up to 14 days of incubation: The carrier of the virus is contagious even before the appearance of the first symptoms: This is why the executive announces that the confinement should last at least 2 weeks before seeing the evolution of the figures of the disease.	**Republic President (Emmanuel Macron)** Health defense council: Group of 10–20 politicians, senior officials, and specialists. Put in action by Emmanuel Macron, it is the principal crisis management tool where the reunions are called almost weekly to take the important executive decisions. Questions can be addressed to ministers, but in the end, the decision is always up to the head of state. Parliament: Vote health emergency state and its extension, control governments actions with commissions of inquiry (e.g., Senate Commission of inquiry, National assembly commission of inquiry, both created in the COVID-19 context), vote the budget for social security. **Prime Minister (Jean Castex)** In charge of the decisions on restrictions and obligations like the switch to health emergency state, lockdown, and curfew. **Health Minister (Olivier Veran)** Put in place the Orsan plan (exceptional system for organizing care in a crisis situation.) Public Health: Tracking of contact people, research, data collection, a daily publication of COVID-19 data, management of the stock (medicines and material), centralization of information about the virus variants. Regional Health agencies: First line of COVID-19 battle: Implementation of the national health policy, organization of patients care, surveillance and prevention, supervision of vaccination logistic, implementation of testing, and increasing health facilities capacity. **4. Mayors** In charge of restrictions and obligations, implementation of curfews with local decrees and vaccination interlocutors. In general, a highly centralized process.	Transmission: it is a respiratory virus contained in sinuses or larynx secretions that can be transmitted in the form of micro-droplets when a patient coughs or sneeze. The droplets do not spread beyond one meter and do not float in the air, according to experts. The chirurgical mask can help limit the spread between patients and people in front of them but is not recommended for the general population. Recommended barrier gestures include regular hand wash, coughing or sneezing into the elbow, avoid shaking hands, and hugging with other people. Incubation: COVID-19 can be transmitted before the first symptoms appear, and asymptomatic people can also transmit the disease. Statistics known: 20% of patients are hospitalized (but factors aggravating the disease are still unknown, but a study shows that the elderly and people who already have other illnesses are more at risk) Early partial assessment: 3.9% of deaths among patients (in the world). An infected individual can contaminate 2 to 2.5 other people. Up to 14 days of incubation, but generally varies from 3 to 5 days.	“We are at war,” was repeated 6 times by Emmanuel Macron on March 15, adding that general mobilization was needed in regard to the total lockdown. “War is a powerful metaphor. It is an effective, immediate, and emotive tool to communicate urgency to the general public” (Caso, 2020).
				
Sweden	March 2: “People in the Stockholm region return to work and school after the half-term winter break, spent by many in the Alps, where the virus is spreading quickly” (The Local, 2021). March 11: Sweden reports its first confirmed death from the coronavirus, on the same day the World Health Organization classifies the global outbreak as a pandemic. March 16: Social isolation of cases and ban of large gathering, Sweden has 1,103 cumulative cases. “When asked by a British reporter what evidence he had to justify Sweden's strategy, he asked rhetorically what evidence other countries had to justify theirs (PM 2020, April 3). Indeed, Tegnell declared himself “very skeptical of lock-downs” in general. “We can’t kill all our services,” he reasoned. “And unemployed people are a great threat to public health” (MailOnline 2020, April 4). In a radio lecture in June (Tegnell 2020), he called his agency's coronavirus strategy a “classic” response to an epidemic. The rest of the world, he had thought, seemed to have “gone mad.” “Restrictions on people's behavior would only delay this spread, not prevent it.” (Andersson & Aylott, 2020, p. 5) Sweden's approach to dealing with the pandemic was influenced by the culture and the importance of trusting each other (Andersson & Aylott, 2020; Yan et al., 2020). Sweden has implemented only measures that have proven to be effective, which is why more drastic measures adopted by other countries, such as closing the borders and forced lockdown, were not implemented in Sweden, as their effectiveness can be questionable from an evidence-based perspective.	The Swedish Government was informed and advised by several actors from different domains. The Public Health Agency (PHA) used an evidence-based approach to formulate measures, while the National Board of Health and Welfare has developed national guidelines and was concerned about the capacity of health facilities. The Swedish Civil Contingencies Agency was mandated to support collaboration between the different actors and analyze the consequences of the pandemic on both short- and long term, particularly its impact on society. Other actors such as the Department of Education, Swedish universities, the Ministry of Foreign Affairs, the Medical Product Agency, the WHO, the European Union, and the European Centre for Disease Control have all influenced and contributed to the Swedish COVID-19 strategy. In general, a decentralized process with a strong say to local authorities and constant population involvement.	Decision was taken at the same time as the French lockdown so Sweden had in hand the same knowledge about the virus. Recognition of limited knowledge, prudent and careful approach with attention to vulnerable people.	“For many Swedes, their state epidemiologist has embodied a rational approach as other countries have sacrificed science to emotion” (Milne, 2020). There is a Swedish model, mostly based on decentralization and local democracy, and Hostede's study shows that Sweden scores low on hierarchical distance, uncertainty avoidance and high on indulgence. All of these make Swedes less insecure when facing change, than say French citizens.

### Stage 1: Impressive Events Build Emotions

The process starts with the news of a major lockdown in China. The media outlets report that the lockdown is in response to a viral epidemic, and the culprit, the SARS-CoV-2, is named. Decisions in China are dramatic, interpreted casually by media commentators as a major epidemic, which could spread to the rest of the world (Vignette 1 below).

#### Vignette 1: The Chinese events: A mirage?

China, probably for cultural and demographic reasons, has been the epicenter of most recent pandemics. The Asian flu, H2N2, in 1958, the H3N2 avian flu in 1968, and in 1997, the SARS-CoV-1 in 2002, and several others have all originated in China. Flynn and Lenaghan (2007) suggest that in Chinese culture, meat quality is associated with its freshness. This increases the proximity between humans and animals and the possibility of virus infections spreading from animals to humans.

In 2002, the Chinese government was caught off guard by SARS-CoV-1, a coronavirus infection, supposedly more deadly than SARS-CoV-2 though less contagious. It decided to put in place a public health response system. Four emergencies were defined: (1) major epidemics; (2) mass illness with unknown causes; (3) large-scale food or professional poisoning; and (4) other important public health-related events, including infectious spills from research labs. The system has three emergency levels, yellow, orange, and red. Two intervention levels are involved, provincial and national. The national level is involved in the highest emergency. In such a case, a national crisis command (NCC) is formed under the authority of the State Council. It includes the Prime Minister or deputy, armed forces’ high officers, and representatives of 32 ministries or state commissions. Similar arrangements exist for each province, city, and county. All territorial levels are involved in response to a national emergency (Liang & Xue, 2004). Four severity levels of intervention are mentioned, the first being the highest.

The SARS-CoV-2 pandemic appeared for reasons described as similar to the 2002 SARS1. On November 17, 2019, a 55-year-old man of Hubei Province was diagnosed with a viral pneumonia infection, believed to be the first SARS-CoV-2 case (AP News, 2021; Government of China, 2020; Tardáguila & Chen, 2020; Taylor, 2021; Xinhua, 2020). It was somewhat disregarded until mid-December when other cases appeared in Wuhan, the capital city of Hubei. On December 31, special attention was given to the 27 cases reported in Wuhan, and the WHO was informed of the possibility of a new virus with pneumonia symptoms. On January 1, 2020, the NCC^4^ was put in place. For unclear reasons, discussed later, it decreed the first level of epidemic severity, the highest. The U.S.'s CDC was informed. Testing was put in place. On January 10, the genome of the new virus was made available to the WHO and all countries. Testing kits were developed by research institutions, including the Wuhan Institute of Virology (WIV). The first death was reported on January 11. Social management measures were strengthened, body temperature was monitored at rail stations and ports, and crowd gathering was restricted. On January 17, seven inspection teams from the NCC began a tour across provinces to instruct about epidemic prevention and control at the local level. On January 20, President Xi Jinping issues important notices, instructing CCP and government to give top priority to the epidemic. At that point, there were 198 cases and three deaths. On January 23, Wuhan, a major city of 11M people in the Center (Huazong) of China, was put under tight quarantine, followed soon by the whole of Hubei Province (AlTakarli, 2020). The following day, 346 medical teams composed of 42,600 medical workers and 965 public health workers from across the country and the armed forces were dispatched to Wuhan. The NCC asked all provinces to take prevention measures, corresponding to local emergency levels. The population was warned to forgo the celebration of the Rat Chinese New Year, a first in peace times. From 23 to 29 January, coordinating mechanisms were put in place and all China was behaving as one organization, dealing with pandemic-related economic, social, and medical issues. For example, 15 provinces or autonomous regions, of which Shandong, Anhui, Hainan, and Tianjin, were to ensure supplies of food, and medical necessities. Zhejiang, Jiangsu, and other advanced provinces provided online government services. In addition, each territorial level made decisions compatible with those of higher levels. On February 2, one of the two new hospitals dedicated to combatting the epidemic opens only 10 days after construction began.

Technology used was impressive, and major firms collaborated. Beidu provided the geolocation system (GNSS) needed to manage the now pandemic. Gaofen provided satellite surveillance. Combining data handling from TFSTAR, a second-generation artificial intelligence satellite, and geocoding, a precise visualization was made available to locate cases and virus spread. To make products available and distribute them safely, a large number of robots and autonomous vehicles were used, handled by such technology giants as Alibaba, Tencent, and Baidu. Drones and facial recognition were put in place to ensure control. And all the companies also provided support to research conducted to decode the virus and follow its many mutating forms.

Patients diagnosed with an infection were also taken care of with four levels of health care, according to the level of pain experienced. Apart from severe cases, all the others were sent home and treated through online medicine, described as being advantageous for both patients and health staff (Song et al., 2020). Most important, the NCC provided an impressive level of coordination to this multiplicity of actions. China's actions made the news of world media reports on Wuhan. The scale of the undertaking and resources used had a real imprint all over the world.

### Stage 2: Fear Grows and Takes Over

Fear sets in, fueled by Wuhan's massive response and two other factors. First, from their previous studies and Chinese information, epidemiology experts derived full descriptions of the virus, highlighting its dangerous characteristics. Pressed by the media, they pulled the emergency alarm, convinced that this was the next most important pandemic since the Spanish Flu. Then, worrying news came from Northern Italy, where high virus casualties were recorded in Bergamo. The level of fear increased further (Vignette 2 below).

#### Vignette 2: The process by which fear spreads: misleading information and one-sided expertise

In France, on January 24, the first three cases were diagnosed in Paris and Bordeaux. The Regional health agencies (ARS) began screening and investigating infection cases. On January 27^,^ a Health Crisis Center (HCC) was set up. Two weeks later, a Health emergency organization plan (ORSAN) was activated. On February 24, 2020, 70 health facilities joined the existing medical emergency service (SAMU) as an alternative first-line care to infected patients. A few days later, the President hosted a defense council and an exceptional council to respond to the coronavirus, to signal a second level in the emergency plan. From March 11 to 17, a radical lockdown was implemented, and a multi-minister crisis unit was activated. On March 16, the President announced: “We are at war!” The lockdown was terminated on May 11 and two other lockdowns followed to keep the country under health emergency until May 2021 (Élysée, 2020; HuffPost, 2020; Le Monde, 2020; Vie Publique, 2020a, 2020b).

Early 2020, the French government was watching what was happening in China, and like the whole world was impressed with the massive use of resources used to stop the coronavirus epidemic. As for SARS-CoV-1, it was assumed that the epidemic would be controlled by China's measures. An early assumption was also that the SARS-CoV-2 Virus was less lethal. Late in February, surprising news from Italy showed that the virus was very active in the Bergamo region, with a high mortality rate. No clear reasons were given, for neither the contagion speed with which the virus was spreading nor the high level of fatalities. Anxiety increased throughout Europe.

Videos of conferences circulating on the web, given by epidemiologists and virologists, provided detailed descriptions of coronaviruses. The messages were alarming. The virus seemed deadly, although no data about actual fatalities were available. The classic response to viral infection was repeated by many. To stop a virus for which there is no known cure, isolating cases or confining populations were the only two possibilities (Ferguson et al., 2020).

The three events, China's massive response, Italy's difficulties in Bergamo, and experts’ normative conclusions, increased the level of anxiety in most of Europe, particularly in France. The Chinese situation was hard to understand because the real intent of the Chinese government was unknown. Whether the measures were part of an exercise to prepare for a biological war or a response to this SARS-Cov-2 was unclear. The Italian situation was also peculiar. In the Bergamo region, a large population of Chinese expatriates was involved in manufacturing businesses, and probably a reason for the epidemic flare-up. Also, unknown then, more than 99% of the reported deaths were older adults with multiple debilitating ailments. Several conferences by world epidemiologists repeated what was common wisdom that viruses are not well known and can be dangerous.

Coronaviruses were known as a particularly active influenza agent. A few years before, other coronaviruses, the H1N1 and the MERS were devastating, particularly in the Middle East. The fear of losing control was reinforced by WHO's announcement that the Chinese epidemic was a pandemic, spreading simultaneously in several countries. The question was, how to respond? Faced with large uncertainties and no convincing justifications but fear of catastrophic public health consequences, the French government responded with a radical decision to lockdown the whole country, a peacetime first. In a demonstration of institutional isomorphism, the French decision was soon imitated by all governments in Europe, Canada, Australia, North Africa, and many others all over the world. The sole and “lonely” exception was Sweden.

### Stage 3: Isomorphic Lockdown

In France, the decision was quickly made to lock down the whole population with limited knowledge about the virus's real effects. Every business and school were directed to close, and individuals to stay home (Vignette 3 below).

#### Vignette 3: The French radical response

The French health system is a highly centralized public service. The government tightly controls the hirings and budgets of all health facilities, except those of the private sector, a small minority. In the early 2000s, it was seen as one of the best in Europe. But, under budgetary stress in the following decade, its performance was steadily declining (Bigot, 2020; Monconduit, 2016).

Seventeen days after the first infection, on March 10, the French government put in place a scientific committee to advise about possible actions. The committee was composed of 13 experts, most of them researchers in disciplines relevant to virus infection. Another committee of 12 researchers or practitioners in the health sciences was set up to advise about providing care to those infected.

On March 12, 2020, President Macron announced that kindergartens, schools, and universities would be closed starting from March 16. He also announced a lockdown of the whole country, closing businesses and restricting people's ability to move. “We are at war!” was repeated 6 times in his speech to emphasize the perceived dangers (Lemarié & Pietralunga, 2020). War measures contradicted the French constitution by injuring individual rights, particularly citizens’ freedom to move (Reicher & Stott, 2020). This would lead to a bumpy relationship between government and population. Thirty-four percent of the latter expressed mistrust of their government (OpinionWay-Ceviprof, 2020).

The haste with which the government reacted was obvious when the bureaucracy was unable to follow. First, the response contradicted many of the rules and procedures put in place over time (Ruimy et al., 2020). Second, many essentials, such as procedure masks, were not available and had to be imported under emergency from China, thus delaying a reliable response. Also, no clear outlook was provided, except that the virus should be stopped at any cost.

The government and its advisers assumed that there is a clear relationship between virus spread and lockdown, which is not the case. The emergency situation was expected to last 1 or 2 months at most, but this prediction neglected the reactive effect of people's fear and their possible resentment of any actions which seemed to undo “needed precautions.” On May 7, the PM announced the end of the total lockdown. But the pandemic pursued its course, and under pressure by the second wave of infections, another lockdown was decided in October, released in December, and then again reinstituted in March 2021 to face the third wave.

Focused on academic health and epidemiology theories, government experts neglected populations’ behavior, which led to resistance among various population segments, and a recurring viral infection spread. The situation was similar all over Europe, Canada, and many other places. The U.K. government attempted to follow a different course of action but did it too casually. The political backlash to an infection spike was such that it came back to what others were doing. The United States was divided and hesitant, and Sweden followed a different strategy (Davies & Roeber, 2021; Drake, 2020; VanDusky-Allen & Shvetsova, 2021). In early 2021, it was clear that the only way out of the cycle of fear and constraints was a vaccine.

#### Vignette 4: The Swedish response: Moderation and effectiveness

The second turning point during stage 3 is the importance of exaggerations and isomorphic behavior as the crisis started feeding itself. Country governments scrutinized by the media and fearful of political costs had all to follow. A little miracle was Sweden's decision to do differently, the exception to what became a rule.

The first SARS-CoV-2 infection in Sweden was recorded on January 31, 2020, in Jönköping. The health structure in Sweden is decentralized. Regional public health agencies are responsible for measures in response to a pandemic. The county chief health officer (CHO) is responsible for the prevention and supervision of health service providers. Municipalities must care for seniors and people with disabilities. A national agency oversees, coordinating, and providing support where necessary. Local authorities, counties, and cities have tax power to fund health expenses. In general, these various agencies’ approach is to seek collaboration from targeted populations rather than use coercive means.

The Swedish infectious disease system was well in place when the pandemic arrived. Public health authorities had a special plan for emergencies. As the Swedish constitution does not allow lockdown, except during war emergencies, they ruled it out, and sought the population's support to fight the pandemic. The response plan put in place was simple. Vulnerable people (elders with associated ailments in particular) were first protected. Visits to elderly care homes were restricted, and so were gatherings of more than 50 people. Post-obligatory schools were closed, and distance teaching was recommended. Everything else, including obligatory-level schools, remained open, with some adaptation measures. For example, stores had special elderly shopping hours (Moisan, 2020). And finally, the population was asked to be cautious and given advice on physical distancing, strict hygiene, and confinement measures in case of infection symptoms (Tegnell, 2021). International frontiers remained open.

Despite international criticisms, the Swedish health authorities remained steadfast and consistent. They regularly explained the situation to the population and sought its input. They attempted to reduce fear, even in the face of international turmoil. They recognized in their international communications the limits of their knowledge and their willingness to adjust where necessary. Local health authorities actually made regular adjustments to meet the pandemic evolution. Sweden comes out of the crisis relatively less injured than other nations. Life seems to come back to normal with limited damage to public health or the economy. The fight against the disease was effective and widely supported by the population, and the WHO suggested that the Swedish model was probably the best way to fight a pandemic.

### Stage 4: An Intractable and Prolonged Crisis

#### Vignette 5: A crisis with no natural end

Institutional isomorphism throughout the world was irresistible. With the media's help, solutions appeared to be standard. When a country locked down, all the others felt obliged to follow. When a country took some initiatives that were applauded by the media, all others followed. For example, when a country ordered procedure masks, all did and sometimes fought for access to supplies. When a country announced that masks for the whole population were not necessary, soon the others followed. When masks were available and were declared necessary somewhere, they became a norm everywhere. When the vaccine was released, the media-driven standard was the speed of the vaccination effort, which led to a free-for-all behavior to secure supplies.

People's fears about the pandemic and governments’ fear of losing the population's support drove a never-ending exercise of confinement and deconfinement. A year and half after the first lockdown, there were still country leaders, and health authorities fixated on “flattening the curve,” and, in general, doing better than the neighbors. The cost of locking down whole populations was staggering. Decisions to lockdown led to spending well over 16,000 billion dollars by the United States alone. In contrast, according to a study, it takes 330 billion dollars to eradicate hunger in the World by 2030 (The Guardian, 2020). This looked like a general failure of leadership and people management. Along with China, which declared the virus vanquished 2 months after the Wuhan flare, the sole well-organized contrarian, Sweden, was in much better shape.

## Discussion and Conclusion

Where does our study lead us? Many social science scholars have attempted to understand better the ramifications and dynamics of the coronavirus pandemic, a genuine historical event. These scholars have mainly focused on the side-effects of public health policies against COVID-19 (Lansiaux et al., 2021), the social policies put in place to help citizens during the pandemic (Hick & Murphy, 2021), how the COVID-19 outbreak affects welfare systems (Greve et al., 2021), the variations in state responses to COVID-19 (Maor & Howlett, 2020), the disproportionate response to COVID-19 (Maor et al., 2020), as well as the fear of COVID-19 among individuals worldwide from a psychological and mental health perspective (Ahorsu et al., 2020). However, what is missing is a finer understanding of how individual emotions quickly turned into collective fear and generated a cascading, isomorphic set of exaggerated responses in most countries. Collective health fear, fed by politically fearful leadership drove out the early search for reasonable courses of action. Unable to question the massive decision to lockdown, the whole crisis was to manage its consequences.

In many ways, we already know quite a bit about the role of emotions in disturbing rational decision-making and behavior. In a pathbreaking study, Elias Canetti (1984) provided a crowd behavior theory to explain why Germany had fallen under the spell of Nazi ideology and organization. He proposed that fear, and in general, emotions could lead to irrational crowd behavior. Since then, it has been well demonstrated that emotions do affect collective decision-making, generally overlooking facts and pushing for impulsive actions (Andrade & Ariely, 2009; Angie et al., 2011; Fenton-O’Creevy et al., 2011; Neumann, 2017). In the SARS pandemic, the ability to understand what was going on was severely constrained by the one-sided advice that governments sought. “Performative scientism,” or the willingness to label “science” any opinion which supports official positions (Muller, 2021), was a source of judgment imbalance. This is like allowing the finance or marketing department to make strategic decisions in a firm, disregarding other functions’ input. Misjudgments and drift are likely (Andrews, 1971).

Our study extends this body of research by emphasizing the interactional nature of policy overreaction during situations of emergencies. In fact, we illustrated how policymakers in all three countries are themselves in constant interaction with citizens, scientists, and international institutions. Where emotions are strong, these interactions can amplify decision-making biases, especially in contexts where rational facts are limited and themselves subject to constant debate. Our study thus expands our understanding of emotions, especially fear, in the decision-making process in situations of uncertainties and emergencies. Furthermore, our study also adds to our understanding of policy overreaction by suggesting the importance of preparedness to be able to moderate the effect of emotions in major natural events and crises. If we scrutinize the events, we can see that Chinese authorities had a level of preparedness for the pandemic unseen anywhere else. Their effectiveness was impressive, even if we discount the effect of people's docility in the face of centralized and authoritarian decision-making. In the aftermath of the SARS 1 in 2003, the Chinese government built a comprehensive emergency response apparatus, waiting for a real-life test.

Several reasons could explain the Chinese level of response preparedness. First, China has the World's largest population. In health or security emergency cases, the ability to respond is reduced by the large volume of communication and action to be taken, and the level of coordination required. Also, the Chinese authorities have been alarmed by the geopolitical aggressiveness of U.S. policies toward China. The American government issued two policy papers, making China a strategic rival and possibly an enemy. The Chinese government responded with its own policy paper on “National defense in a new era,” where the U.S. policy is seen as a threat, “It has […] significantly increased its defense expenditure […] and undermined global strategic stability^5^” (Werner, 2019). A nuclear confrontation is unlikely, but a biological war is feared because of its stealth potential. Therefore, it is plausible to assume that the Chinese well-organized response to the pandemic was part of drilling the population to respond to biological aggression.

Sweden was exposed to the same stimuli coming from other nations, including China. The geopolitical threat was minimal, despite occasional tensions with the Russian neighbor. Also, Sweden is decentralized and serious about popular democracy. Locking down populations is seen as a last resort. Trusting people's willingness to protect themselves and contribute to local safety, central authorities refrained from making radical decisions. Nevertheless, they decided to attract people's attention, like protecting the vulnerable, closing post-obligatory schools, limiting crowd gatherings, yet reducing fear through competent communication. The Swedes generally supported these policies, despite international media pressures to imitate other countries.

France can be considered a middle power, still important in Europe, along with Germany and the U.K. But France's means are limited, and the nation is obliged to navigate between traditional allies, such as the United States, and rising nations like China and Russia. This delicate attempt at balance puts the country leaders on edge, with constant worries about national standing. The need to be geopolitically well-positioned pushed France to play difficult games (e.g., Libya and African Sahel difficult involvements). Therefore, it is reasonable to expect France's leaders to be more emotional than Sweden's and jump to conclusions faster, even in the face of limited information. The French government's highly centralized and professionally biased organization used a war narrative and coercion to fight the pandemic. The population's resistance to the lockdown has been constant. Results were well below expectations. The French experience was imitated in countless other countries of the world, in particular Western democracies.

These situations point at a generalized failure to deal with a pandemic of moderate severity. Most of the failure comes from policy overreaction, fueled by emotions and institutional isomorphism, instead of fact-based decision-making. The effect of emotions should be a concern, especially in situations of grave danger or emergency. President Bush, under emotion, has gone to war in Afghanistan and Iraq, called “the never-ending wars” by President Biden. In contrast, President Kennedy's warry of emotions has averted nuclear war during the Cuban missile crisis (Allison, 1971). Thus, an ability to respond rationally to an emergency is a critical, disaster-averting measure of leadership and organizational performance.

In complex and uncertain situations, policy decision-making is difficult and of momentous consequences (Geyer & Cairney, 2015; Mueller, 2020). While overreaction in policymaking under emergency is explainable (Desch, 2008; Maor, 2014, 2018), it is nevertheless dangerous for society. The willingness of governments to show steadfast leadership in a complex crisis may lead to emotional overreaction and damage a country's future well-being (Reicher & Stott, 2020). Based on our study, a concatenation of five major cures can be used to control emotions and institutional isomorphism or limit their effects:

1. **Fact-based management:** Looking for facts should be the first concern in situations of emergency. International influence and its isomorphic effects are strong. It is important to ensure that others’ solutions are appropriate given the local context. But doing so does not prevent from taking speedy measures to protect those people most vulnerable to the emergency effects.
2. **Incremental decision:** The decision-making process most effective in an emergency is piecemeal, making small decisions, which can be quickly reversed if need be. Small decisions are also a way to learn about the phenomena policymakers are faced with. In contrast, large decisions are a one-way street. If wrong, there is no return possibility.
3. **Decentralize response decisions:** Most of the effects of a pandemic are local. Broad responses are bound to be unacceptably harsh for most people and wrong, which would lead to people's resistance and failure. But local decisions have to be coordinated and supported by a well-designed central office.
4. **Ensure open communication and value popular input and support:** In the face of danger, populations are generally collectively obedient and reasonable. Inconsistency and a lack of transparency could lead them to revert to a free-for-all individualistic behavior. To remain united in the face of threat, they need clear information, courageous leadership, and candid debate. When properly guided, people can contribute important information and effective local measures.
5. **Build balanced structures** to inquire about the emergency and to guide the response to its effects. Advisers provide key inputs and play an important complementary managerial role. They should be diversified and inclusive of all important aspects of the emergency. These are often hard to provide in situations of emergency. The example of China, despite its authoritarian character, reveals the importance of preparation. The example of Sweden reveals the power of modest and sensible leadership in fighting fear, building confidence and mutual trust, and popular support. Both have been able to overcome irrational fear and institutional pressures to build seemingly reasonable responses.

## Notes

1. On April 14, 2021, according to the NYTimes, President Biden announced that the USA would definitely evacuate Afghanistan, with no preliminary conditions, on September 11, 2021, the 20th anniversary of the invasion.

2. It was later shown that the Italian high mortality among the Etruscans of Bergamo was related to some kind of viral interference, because these populations, generally resistant to vaccination, had received two important vaccines (Flu and Meningitis) in quick sequence, in October 2019 and early 2020.

3. The name of this group varies in public translations. Other names are National Health Commission (NHC) and Central Leading Group (CLG).

4. This is the title of the White paper final section.
